# Reactive Oxygen Species and Gibberellin Acid Mutual Induction to Regulate Tobacco Seed Germination

**DOI:** 10.3389/fpls.2018.01279

**Published:** 2018-10-02

**Authors:** Zhan Li, Yue Gao, Yuchan Zhang, Cheng Lin, Dongting Gong, Yajing Guan, Jin Hu

**Affiliations:** Seed Science Center, College of Agriculture and Biotechnology, Zhejiang University, Hangzhou, China

**Keywords:** tobacco seed, germination, H_2_O_2_, GA, ABA, homeostasis, signal transduction

## Abstract

Seed germination is a complex process controlled by various mechanisms. To examine the potential contribution of reactive oxygen species (ROS) and gibberellin acid (GA) in regulating seed germination, diphenylene iodonium chloride (DPI) and uniconazole (Uni), as hydrogen peroxide (H_2_O_2_) and GA synthesis inhibitor, respectively, were exogenously applied on tobacco seeds using the seed priming method. Seed priming with DPI or Uni decreased germination percentage as compared with priming with H_2_O, especially the DPI + Uni combination. H_2_O_2_ and GA completely reversed the inhibition caused by DPI or Uni. The germination percentages with H_2_O_2_ + Uni and GA + DPI combinations kept the same level as with H_2_O. Meanwhile, GA or H_2_O_2_ increased GA content and deceased ABA content through corresponding gene expressions involving homeostasis and signal transduction. In addition, the activation of storage reserve mobilization and the enhancement of soluble sugar content and isocitrate lyase (ICL) activity were also induced by GA or H_2_O_2_. These results strongly suggested that H_2_O_2_ and GA were essential for tobacco seed germination and by downregulating the ABA/GA ratio and inducing reserve composition mobilization mutually promoted seed germination. Meanwhile, ICL activity was jointly enhanced by a lower ABA/GA ratio and a higher ROS concentration.

## Introduction

Seeds are important genetic resources for sustainable agriculture and agricultural economy. Successful germination and seedling establishment are the foundation of agricultural production (Bentsink and Koornneef, 2008). It has been proposed that seed germination is mediated by gibberellin acid (GA) that promotes germination completion. Abscisic acid (ABA) has antagonistic effects on GA in inducing seed dormancy. The dynamic balance of ABA and GA, resulting from their synthesis, catabolism, and signaling pathway, is crucial for seed germination (Razem et al., 2006; Seo et al., 2006; Weiss and Ori, 2007; Topham et al., 2017). Up to now, the molecular mechanism of interaction between ABA and GA during germination remains largely elusive. It is well known that *NCED* (encoding 9-*cis*-epoxycarotenoid dioxygenase) is a key gene in ABA biosynthesis, and *CYP707A* (encoding ABA 8-hydroxylases) is the essential gene in ABA catabolism (Footitt et al., 2011). ABA insensitive 3 (ABI3) and ABA insensitive 5 (ABI5), the central ABA signaling components, serve as the final downstream repressors of seed germination in the ABA signaling pathway (Carles et al., 2002; Lim et al., 2013; Liu et al., 2013). *GA20ox* (encoding GA 20-oxidase) and *GA3ox* (encoding GA 3-oxidase) are critical genes during bioactive GA biosynthesis and *GA2ox* (encoding GA 2-oxidase) transfers active GAs to inactive GAs. RGL2 (repressor of GA-like protein), one of plant growth repressor DELLA proteins (DELLAs) (Zentella et al., 2007; Golldack et al., 2013), is considered the vital repressor of seed germination (Piskurewicz et al., 2008; Stamm et al., 2012). Gibberellin-insensitive dwarf protein (GID), a receptor of GA signaling, could interact with GA and eliminate the inhibition of seed germination by triggering the degradation of RGL2 (Cao et al., 2006; Griffiths et al., 2006).

Seed reserve composition mobilization is an important requirement for germination as well as subsequent plant growth and development (Bau et al., 1997; Kupidłowska et al., 2006). It should be noted that the lipid content of tobacco seed is up to 40% by weight (Giannelos et al., 2002). Isocitrate lyase (ICL, EC 4.1.3.1) is a key enzyme during the glyoxylate cycle that plays a major role in affecting net gluconeogenesis from lipid metabolism (Borek and Nuc, 2011). Generally, seed germination and seedling establishment are restrained by inhibiting ICL activity, as demonstrated in oilseeds such as sunflower (*Helianthus annuus* L.), coffee (*Coffea arabica* L.), and canola (*Brassica napus* L.) (Baleroni et al., 2000; Kupidłowska et al., 2006; Selmar et al., 2006). In addition, phytohormones ABA and GA are widely known to control the mobilization of lipids by inhibiting or inducing ICL activity and the responding gene expression in rice (*Oryza sativa* L.), wheat (*Triticum aestivum* L.), and barley (*Hordeum vulgare* L.) (Eastmond and Jones, 2005; Penfield et al., 2006).

Reactive oxygen species (ROS) act as regulators of growth and development, programmed cell death, hormone signaling, and responses to biotic and abiotic stresses (Breusegem et al., 2008). In seeds, ROS play a key role in various events, such as maturation, ripening, aging, and germination (Bailly et al., 2008; Müller et al., 2009; Barba-Espín et al., 2011). Plasma membrane nicotinamide adenine dinucleotide phosphate (NADPH) oxidase, the pivotal enzyme involved in ROS generation, regulates germination of barley seeds (Ishibashi et al., 2010). Hydrogen peroxide (H_2_O_2_) is considered as the ROS messenger for long-distance transport in cells (Moller, 2001; Möller and Sweetlove, 2010), the stimulation of downstream transcription, and the activation of mitogen-activated protein kinases (MAPKs) (Neill S. et al., 2002). Exogenous H_2_O_2_ stimulates seed germination of Arabidopsis (*Arabidopsis thaliana* L.), maize (*Zea mays* L.), and sunflower seeds by enhancing GA biosynthesis and ABA catabolism (Maarouf et al., 2015; Huang et al., 2017; Li et al., 2017). Nevertheless, H_2_O_2_ releases embryo dormancy in barley by activating GA signaling and synthesis rather than the repression of ABA signaling (Bahin et al., 2011). GA biosynthesizes in embryo during the germination and induces H_2_O_2_ production in wheat aleurone cells; H_2_O_2_ subsequently acts as a signal molecule to antagonize ABA signaling (Wu et al., 2014). Additional evidence has suggested that GA induces the germination of dormant caryopses by regulating the level of ABA and the ROS-antioxidant status (Cembrowska-Lech et al., 2015). Programmed cell death is induced by GA in aleurone layers also via the regulation of H_2_O_2_ production (Aoki et al., 2014). The application of H_2_O_2_-induced cell death was observed only under GA treatment but not ABA treatment (Bailly, 2013). Although several evidences indicate the crosstalk among ROS, GA and ABA affect seed germination, but the interaction between them remains elusive. Is there a relationship between GA and ROS that mutually induced or maintained homeostasis during seed germination? Similarly, it is unknown how ROS and GA synergistically control ABA levels during seed germination. Moreover, the relationship among ROS, GA, and ABA with ICL activity also has not been analyzed in tobacco seed germination.

Therefore, the purpose of the present study is to examine the interaction mechanism of H_2_O_2_, GA, ABA, and ICL in the process of germination. The effect of diphenylene iodonium chloride (DPI), a NADPH oxidases inhibitor (Morré, 2002), uniconazole (Uni), a GA synthesis inhibitor (Yang et al., 2012), DPI + GA and Uni + H_2_O_2_ on seed germination is investigated. In addition, ICL activity, ABA, GA, H_2_O_2_ contents, and the corresponding gene expressions involved in homeostasis and signal transduction are determined to gain insights on the crosstalk among ROS, ABA, and GA, and to acquire the regulation mechanism of ICL activity, by proposing a comprehensive model of tobacco seed germination.

## Materials and Methods

### Materials

Tobacco seeds, MS Yunyan 97, from Yunnan Tobacco Science Research Institute were used. GA, H_2_O_2_, DPI, and Uni were obtained from Aladdin Company, Hangzhou, China.

### Seed Priming

Seeds were surface-sterilized with 0.5% NaClO solution for 15 min and then washed three times with sterilized distilled water to remove the residue of the disinfectant. The sterilized seeds were poured into a sealed beaker with priming solutions (1:5, w/v) at 20°C in darkness for 24 h, without changing the solutions during the priming process. All primed seeds were air-dried at 25°C for 48 h to the original moisture content. In this study, ten priming solutions including distilled water (H_2_O), 10 mM GA, 50 mM H_2_O_2_, 30 μM Uni, 100 μM DPI, and the corresponding combination priming GA + DPI, H_2_O_2_ + Uni, GA + Uni, H_2_O_2_ + DPI, and Uni + DPI were all carried out.

### Seed Germination

All primed tobacco seeds were germinated on three-layer water-saturated filter papers in germination dishes of 10 cm diameter at 25°C, with a photosynthetic active photon flux density of 250 mmol m^-2^ s^-1^ and a photoperiod of 8 h light (L): 16 h dark (D). In addition, four replications of 100 seeds for each treatment were used. Germinated seeds (radicle emergence) were recorded daily (International Seed Testing Association [ISTA], 2010). Germination percentage (GP) was calculated from the 1st to the 16th day (GP = 100% × G_t_/100, where G_t_ is the total germinated seeds on the tth day. Germination energy (GE) was the GP on the 7th day. Germination index (GI) and mean germination time (MGT) were also calculated on the 2nd, 5th, and 7th days [GI = Σ(G_t_/T_t_); MGT = Σ(G_t_ × T_t_)/ΣG_t_, where G_t_ is the number of new germinated seeds in time T_t_].

### ICL Activity Determination

The ICL activity assay was performed as described by Yanik and Donaldson (2005). The mixture containing 72.5 mM phosphate buffer (pH 6.9), 10.8 mM MgCl_2_, and 10 mM phenylhydrazine in a volume of 1 ml was used. Freshly prepared 1.74 mM isocitrate and protein extracts were then added at 25°C. Phenylhydrazine forms a yellow phenylhydrazone in the presence of glyoxylic acid. The activity was detected by the increase in absorbance at 324 nm using the molar extinction coefficient 𝜀 = 1.7 × 10^4^.

### H_2_O_2_ and Soluble Sugar Content Measurements

Seeds were collected at 0, 24, and 48 h during imbibition. For each treatment, 0.4 g seeds with four biological replications were collected for physiological parameter determination. H_2_O_2_ content was determined according to the method of Doulis et al. (1997), and calculated as μmol H_2_O_2_ decomposition min^-1^⋅g^-1^⋅FW. Soluble sugar content in seeds was measured by using the anthrone colorimetric method (Li and Li, 2013) with a slight modification.

### ABA and GA_3_ Measurements

Seeds were collected at 0, 24, and 48 h during imbibition. Approximately 0.4 g seed powder for each treatment with three biological replications were homogenized with 2 mL of cold acetonitrile, kept at 4°C for 12 h and then centrifuged at 23,000 × *g* for 10 min at 4°C. The supernatant was transferred to a new centrifugal tube and mixed with 1.5 mL of phosphate buffer solution (0.1 M), kept at -80°C for 30 min and thawed adequately at 4°C. The mixture was extracted by 2.5 mL of ethyl acetate three times after the addition of 1 mL hydrochloric acid (0.5 mM). Five milliliters of the ethyl acetate phase was collected after centrifuging at 1500 × *g* for 5 min at 4°C, dried with a nitrogen evaporator, and redissolved in 1 mL mobile phase consisting of methanol and water (1:1, v/v). The collected extract was filtered through a 0.22 mm membrane filter for high-performance liquid chromatography equipped with a 6.0 mm × 150 mm, a 5 mm particle size reverse-phase (C18) column, and an ultraviolet detector (Waters 2487), as previously described (Huang et al., 2017). GA_3_ and ABA peaks were detected by using a SPD-20A (Shimadzu) absorbance detector at 254 nm. The mobile phase consisted of methanol and 0.2% phosphoric acid solution (1:1, v/v) at a flow rate of 0.8 mL min^-1^. Standard GA_3_ and ABA (Sigma Chemical Company) were used for the development of standard curves.

### Total RNA Extraction and Quantitative Real-time PCR (RT-qPCR) Analysis

Total RNA was isolated from seeds using TRIzol reagent (Huayueyang, Beijing, China) and reverse-transcribed using a ReverTra Ace qPCR RT Kit (Toyobo, Osaka, Japan) following the manufacturer’s instructions. Eight genes were involved in the tobacco GA signal: *NtGA20ox1, NtGA20_OX_2, NtGA3_OX_2, NtGA2_OX_1, NtGA2_OX_2, NtGID1, NtGID2*, and *NtRGL2*. Four genes were involved in the ABA signal: *NtNCED1, NtNCED3, NtCYP707A1*, and *NtCYP707A2*. *NtRBOH* (*Nicotiana tabacum* respiratory burst oxidase homolog) and *NtICL* were involved in NADPH oxidase and ICL biosynthesis (**Supplementary Table S1**). RT-qPCR was performed using the Roche real-time PCR detection system (Roche Life Science, United States). Each reaction (20 μL) consisted of 10 μL SYBR Green PCR Master Mix (Takara, Chiga, Japan), 1 μL diluted cDNA, and 0.1 μM forward and reserve primers. The PCR cycling conditions were as follows: 95°C for 3 min, followed by 40 cycles of 95°C for 10 s and 58°C for 45 s. The tobacco Actin gene was used as an internal control. Relative gene expression was calculated according to Livak and Schmittgen (2001).

### Statistical Analysis

Data were analyzed by analysis of variance (ANOVA) using the Statistical Analysis System (SAS) (version 9.2) followed by the calculation of the least significant difference (LSD, α = 0.05). Percentage data were arc-sin-transformed prior to analysis.

## Results

### Uni or DPI Significantly Slowed Down Germination Speed and Reduced GP

**FIGURE 1:**
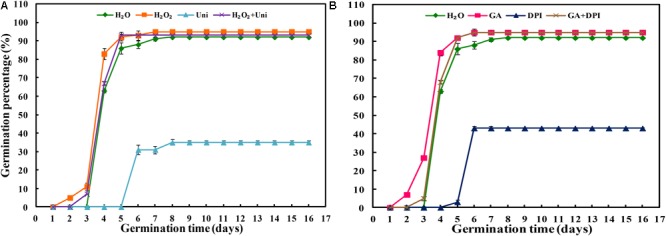
Inhibitor of H_2_O_2_ **(A)** or GA **(B)** decreased seed germination. H_2_O, hydropriming (water priming); H_2_O_2_, 50 mM hydrogen peroxide priming; Uni, priming with 30 μM uniconazole, as GA synthesis inhibitor; H_2_O_2_ + Uni, 50 mM hydrogen peroxide and 30 μM uniconazole combination priming; GA, 10 mM gibberellin acid priming; DPI, priming with 100 μM diphenyliodonium, as H_2_O_2_ scavenger; GA+ DPI, 10 mM gibberellin acid and 100 μM diphenyliodonium combination priming. Vertical bars above mean indicate standard error of four replicates of 100 seeds each for each treatment. Values are mean ± SE (*n* = 4).

Uni or DPI significantly inhibited seed germination compared with other treatments (**Figure 1** and **Supplementary Figure S2**). The GP of Uni was 35% ultimately, and that of DPI was 43%. Seeds treated by Uni + DPI barely germinated, and GP was only 5% until the end of germination (**Supplementary Figure S1**). The seed germination inhibition of Uni or DPI could be reversed by adding GA or H_2_O_2_. Eventually, the GP of H_2_O_2_ + DPI and GA + Uni reached 92%, respectively (**Supplementary Figure S1**). Interestingly, the combination of H_2_O_2_ + Uni or GA + DPI reversed the inhibition effect of Uni or DPI on germination, and the GPs of both were greater than 90%. Seeds treated with H_2_O_2_ or GA started germinating on the second day, and those treated with H_2_O_2_ + Uni or GA + DPI began to germinate on the third day, followed by H_2_O on the fourth day, while seeds treated with Uni or DPI delayed germination until the sixth day of germination when the GP of GA or H_2_O_2_ had already reached 92% (**Figure 1** and **Supplementary Table S2**).

### GA and H_2_O_2_ Treatments Increased Endogenous H_2_O_2_ Content During Seed Imbibition

**FIGURE 2:**
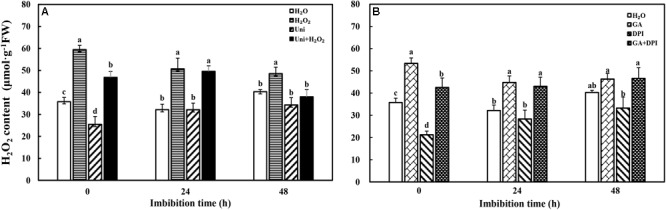
Accumulation of H_2_O_2_ during seed imbibition in response to different treatments. **(A,B)** H_2_O_2_, hydrogen peroxide. Seeds were collected, respectively, at 0, 24, and 48 h during imbibition, and four replications for each treatment at each sampling time were used. Different small letters on top of the bars indicate significant differences (LSD, α = 0.05) among treatments at the same imbibition time. Error bars indicate ± SE of mean (*n* = 4). For additional explanations, please see **Figure 1**.

Exogenous H_2_O_2_ and GA obviously increased endogenous H_2_O_2_ content, while DPI and Uni significantly reduced the content at 0 h of imbibition (also the end time of priming) compared with H_2_O (**Figure 2**). GA + DPI reversed the reduction of endogenous H_2_O_2_ caused by DPI, and H_2_O_2_ + Uni also reversed the reduction caused by Uni, especially from 0 to 24 h.

### ICL Activity and Soluble Sugar Content Decreased Accompanied by Seed Imbibition

**FIGURE 3:**
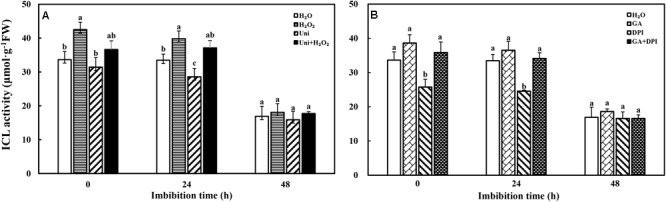
ICL activity decreased accompanied by seed imbibition. **(A,B)** ICL, isocitrate lyase. Seeds were collected, respectively, at 0, 24, and 48 h during imbibition, and four replications for each treatment at each sampling time were used. Different small letters on top of the bars indicate significant differences (LSD, α = 0.05) among treatments at the same imbibition time. Error bars indicate ± SE of mean (*n* = 4). For additional explanations, please see **Figure 1**.

ICL activity had an observable decrease at 48 h that was about half the ICL activity at 0 h (**Figure 3**). The ICL activity of DPI and Uni was notably lower than that of H_2_O at 0 and 24 h, and at 24 h, respectively. The maximal ICL activity was recorded in H_2_O_2_ or GA, followed by H_2_O_2_ + Uni or GA + DPI from 0 to 24 h. Ultimately, all treatments kept the same level at 48 h.

The change trend of soluble sugar was consistent with ICL activity. The main difference was that soluble sugar content dropped significantly at 24 h, which was earlier than 48 h when the changes in ICL activity occurred (**Supplementary Figure S4**). H_2_O_2_ or GA increased the content of soluble sugar at 0 and 24 h, significantly at 24 h, while Uni or DPI decreased the content at 0 and 48 h, significantly at 48 h. H_2_O_2_ + Uni or GA + DPI slightly reversed the effects induced by Uni or DPI at 0 and 48 h.

### Fluctuation of Endogenous ABA and GA_3_ Content During Imbibition

**FIGURE 4:**
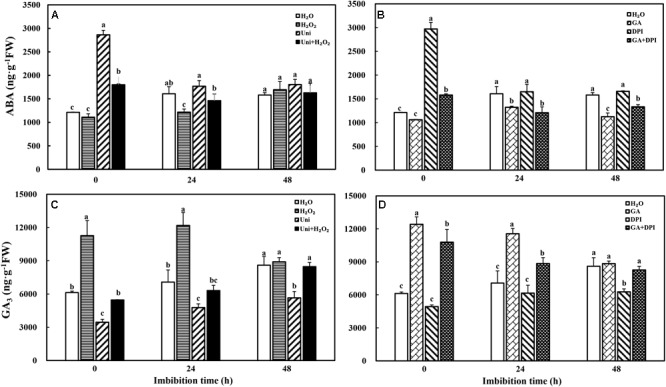
Inhibitor of GA or H_2_O_2_ increased ABA **(A,B)** and GA_3_ **(C,D)** content during seed imbibition in response to different treatments. Seeds were collected, respectively, at 0, 24, and 48 h during imbibition, and four replications for each treatment at each sampling time were used. Different small letters on top of the bars indicate significant differences (LSD, α = 0.05) among treatments at the same imbibition time. Error bars indicate ± SE of mean (*n* = 4). For additional explanations, please see **Figure 1**.

It was rather obvious that both Uni and DPI dramatically induced endogenous ABA accumulation at the beginning of imbibition (**Figures 4A,B**, 0 h). GA decreased ABA content throughout the whole imbibition period, while H_2_O_2_ decreased ABA content at 0 and 24 h. In general, H_2_O_2_ + Uni or GA + DPI counteracted the accumulation of ABA induced by Uni or DPI, of which ABA content was still higher than that in GA or H_2_O_2_, especially at 0 h.

In addition, the rapid and extensive generation of endogenous GA_3_ with H_2_O_2_ or GA treatment was also significantly higher than that with H_2_O treatment at 0 and 24 h. Uni markedly decreased GA_3_ content, which was distinctly lower than H_2_O from 0 to 48 h (**Figure 4C**). H_2_O_2_ + Uni obviously alleviated the decrease in GA_3_ content caused by Uni at 0 and 24 h and stablized at the same GA_3_ level as H_2_O at 48 h. GA_3_ significantly declined at 48 h with DPI treatment (**Figure 4D**). Exogenous GA obviously increased GA_3_ content at 0 and 24 h. GA + DPI slightly decreased GA_3_ content comparing with exogenous GA, which was still significantly higher than H_2_O from 0 to 24 h.

The ABA/GA ratio of Uni and DPI was obviously higher than other treatments at 0 h, which was 2–3 fold as much as H_2_O (**Supplementary Figure S3**), and then decreased sharply at 24 h, when Uni was still significantly higher than H_2_O. The ABA/GA ratio and H_2_O_2_ remained at lower levels during the whole imbibition process.

### ABA Homeostasis and Signal Transduction Were Affected in Different Treatments During Imbibition

**FIGURE 5:**
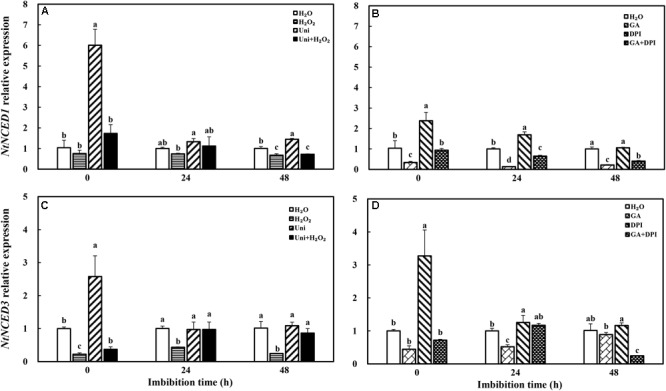
GA or H_2_O_2_ decreased the transcript level of ABA biosynthesis genes *NtNCED1* **(A,B)** and *NtNCED3* **(C,D)** during seed imbibition. Seeds were collected, respectively, at 0, 24, and 48 h during imbibition, and four replications for each treatment at each sampling time were used. Different small letters on top of the bars indicate significant differences (LSD, α = 0.05) among treatments at the same imbibition time. Error bars indicate ± SE of mean (*n* = 4). For additional explanations, please see **Figure 1**.

Both Uni and DPI induced the expression of *NtNCED1* and *NtNCED3*, ABA synthesis genes, at the end of priming (**Figure 5**, 0 h). The *NtNCED1* expression of Uni was 6-fold of H_2_O, and that of DPI was 2.5-fold. Meanwhile, the *NtNCED3* transcription level of DPI was 3.2-fold of H_2_O, and that of Uni was 2.5-fold. H_2_O_2_ significantly decreased *NtNCED3* expression and slightly decreased *NtNCED1* expression, while GA significantly decreased *NtNCED1* expression. At 48 h, H_2_O_2_ + Uni significantly decreased the expression of *NtNCED3*, while GA + DPI decreased the expression of both *NtNCED1* and *NtNCED3*.

**FIGURE 6:**
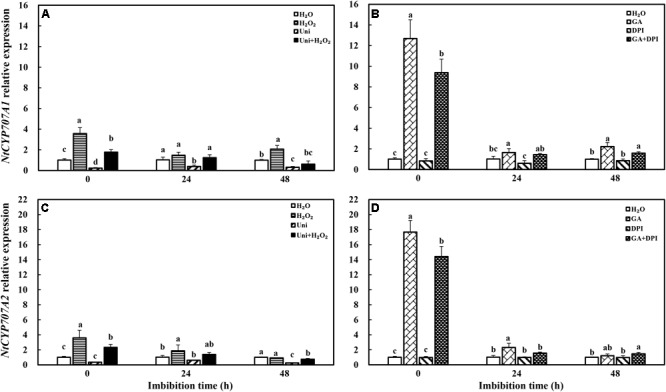
GA or H_2_O_2_ enhanced the transcript level of ABA catabolism genes *NtCYP707A1* **(A,B)** and *NtCYP707A2* **(C,D)** during seed imbibition. Seeds were collected, respectively, at 0, 24, and 48 h during imbibition, and four replications for each treatment at each sampling time were used. Different small letters on top of the bars indicate significant differences (LSD, α = 0.05) among treatments at the same imbibition time. Error bars indicate ± SE of mean (*n* = 4). For additional explanations, please see **Figure 1**.

Gibberellin acid dramatically promoted the transcription level of *NtCYP707A1* and *NtCYP707A2*, ABA catabolism genes, which was, respectively, 12-fold and 17-fold of H_2_O, followed by GA + DPI with 9- and 14-fold of H_2_O, while the effect of H_2_O_2_ was weaker than GA, approximately 3.6- and 4-fold of H_2_O (**Figure 6**). Uni significantly decreased the expression of *NtCYP707A1* and *NtCYP707A2* from 0 to 48 h and at 48 h, respectively, and both remained at a low level during imbibition. DPI had no obvious function on the expression of *NtCYP707A1* and *NtCYP707A2*.

**FIGURE 7:**
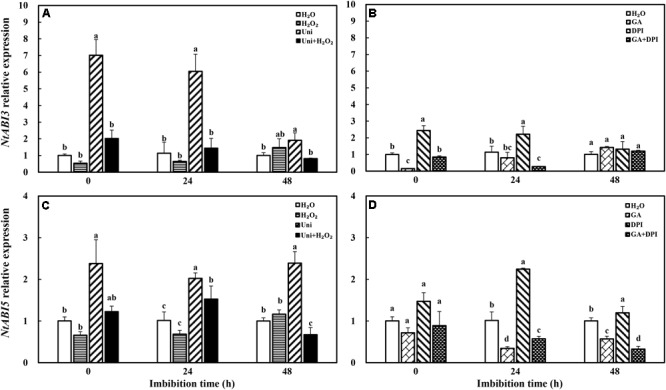
GA or H_2_O_2_ downregulated the transcript level of ABA signaling genes *NtABI3* **(A,B)** and *NtABI5* **(C,D)** during seed imbibition. Seeds were collected, respectively, at 0, 24, and 48 h during imbibition, and four replications for each treatment at each sampling time were used. Different small letters on top of the bars indicate significant differences (LSD, α = 0.05) among treatments at the same imbibition time. Error bars indicate ± SE of mean (*n* = 4). For additional explanations, please see **Figure 1**.

In addition, Uni significantly enhanced *NtABI3* and *NtABI5* expression compared with H_2_O during the whole progress of imbibition (**Figure 7**), and DPI upregulated the expression of *NtABI3* from 0 to 24 h and *NtABI5* from 24 to 48 h. The downregulation induced by H_2_O_2_ was not obvious in *NtABI3* and *NtABI5* similarly to H_2_O_2_ + Uni. GA downregulated the expression of *NtABI3* from 0 to 24 h as well as the expression of *NtABI5* from 24 to 48 h, consistent with GA + DPI.

### GA Homeostasis and Signal Transduction Were Mediated in Different Treatments During Imbibition

**FIGURE 8:**
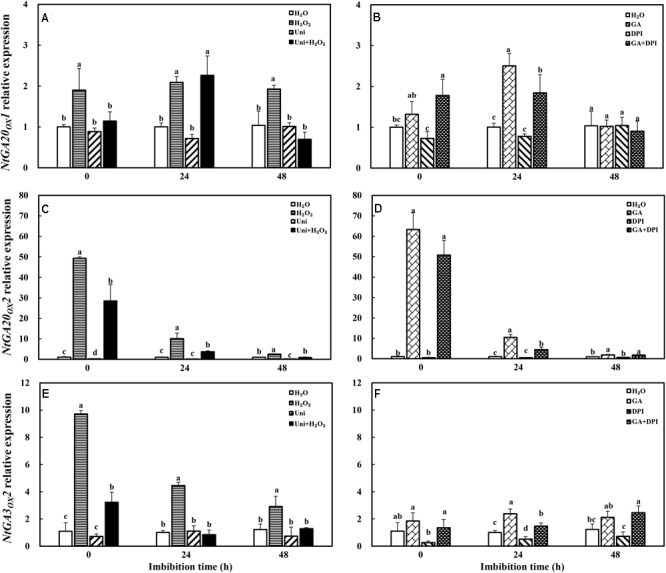
GA or H_2_O_2_ upregulated the transcript level of GA biosynthesis genes *NtGA20_OX_2* **(A,B)**, *NtGA20_OX_1* **(C,D)**, and *NtGA3_OX_2* **(E,F)** during seed imbibition. Seeds were collected, respectively, at 0, 24, and 48 h during imbibition, and four replications for each treatment at each sampling time were used. Different small letters on top of the bars indicate significant differences (LSD, α = 0.05) among treatments at the same imbibition time. Error bars indicate ± SE of mean (*n* = 4). For additional explanations, please see **Figure 1**.

H_2_O_2_ dramatically enhanced the transcription level of GA synthesis genes (*NtGA20_OX_1, NtGA20_OX_2*, and *NtGA3_OX_2*). GA only obviously increased the expression of *NtGA20_OX_2* (**Figure 8**). Uni largely inhibited *NtGA20_OX_2* expression at 0 and 48 h, while H_2_O_2_ + Uni significantly upregulated the expression of *NtGA20_OX_2* from 0 to 24 h, *NtGA20_OX_1* at 24 h, and *NtGA3_OX_2* at 0 h, respectively. In addition, DPI mainly inhibited the expression of *NtGA3_OX_2* and was ineffective in *NtGA20_OX_1* and *NtGA20_OX_2.* GA + DPI obviously upregulated the expression of *NtGA20_OX_2*, followed by *NtGA20_OX_1* and *NtGA3_OX_2.*

**FIGURE 9:**
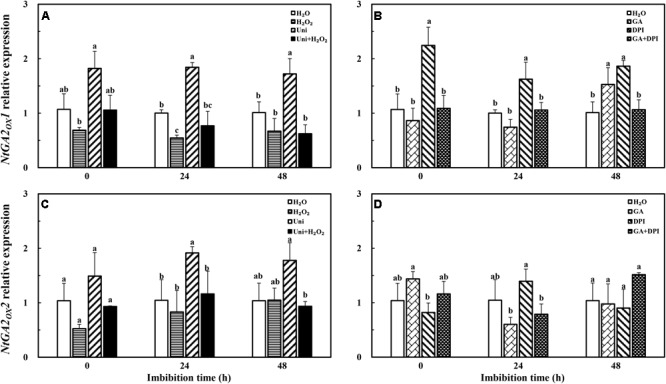
GA or H_2_O_2_ mediated the transcript level of GA catabolism genes *NtGA2_OX_1* **(A,B)** and *NtGA2_OX_2* **(C,D)** during seed imbibition. Seeds were collected, respectively, at 0, 24, and 48 h during imbibition, and four replications for each treatment at each sampling time were used. Different small letters on top of the bars indicate significant differences (LSD, α = 0.05) among treatments at the same imbibition time. Error bars indicate ± SE of mean (*n* = 4). For additional explanations, please see **Figure 1**.

The transcription level of the GA catabolism gene *NtGA2_OX_1* was significantly decreased by H_2_O_2_ at 24 h, but not found in exogenous GA treatment (**Figure 9**). There was no significant difference among GA, H_2_O_2_, and H_2_O treatments with regard to *NtGA2_OX_2*. The expression of the two *NtGA2_OX_* genes was increased rapidly under the Uni condition, and was notably reversed by the addition of H_2_O_2_, meanwhile, *NtGA2_OX_1* expression was upregulated by DPI during the entire imbibition period but inhibited by GA + DPI.

**FIGURE 10:**
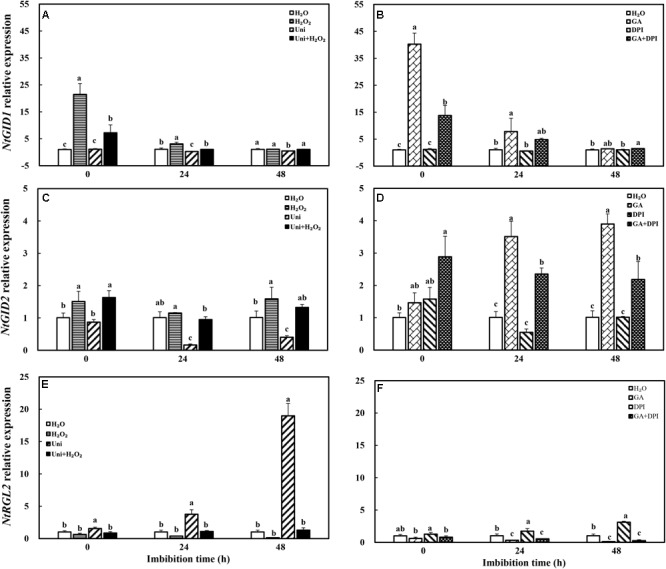
GA or H_2_O_2_ mediated the transcript level of GA signaling genes *NtGID1* **(A,B)**, *NtGID2* **(C,D)**, and *NtRGL2* **(E,F)** during seed imbibition. Seeds were collected, respectively, at 0, 24, and 48 h during imbibition, and four replications for each treatment at each sampling time were used. Different small letters on top of the bars indicate significant differences (LSD, α = 0.05) among treatments at the same imbibition time. Error bars indicate ± SE of mean (*n* = 4). For additional explanations, please see **Figure 1**.

Uni significantly upregulated the transcription level of *NtRGL2* and downregulated the *NtGID2* gene expression, while DPI only influenced the upregulation of *NtRGL2* (**Figure 10**). H_2_O_2_ enhanced the expression of *NtGID1* and *NtGID2* as did H_2_O_2_ + Uni, significantly at 0 h. GA also downregulated the *NtRGL2* expression and upregulated *NtGID1* and *NtGID2*; especially, the *NtGID1* expression was 40-fold of H_2_O at 0 h. GA + DPI obviously enhanced the expression of *NtGID1* and *NtGID2* during the whole imbibition process.

### Treatments Modulated the Expression of NtRBOH and NtICL During Imbibition

**FIGURE 11:**
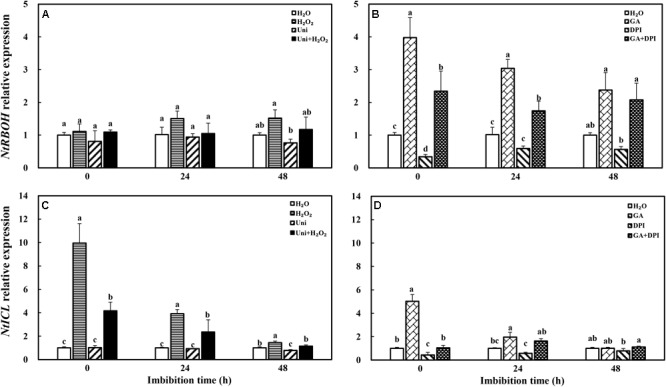
GA or H_2_O_2_ upregulated the transcript level of *NtRBOH* **(A,B)** and *NtICL* **(C,D)** during seed imbibition. *NtRBOH* and *NtICL* were involved in NADPH oxidase and ICL biosynthesis. Seeds were collected, respectively, at 0, 24, and 48 h during imbibition, and four replications for each treatment at each sampling time were used. Different small letters on top of the bars indicate significant differences (LSD, α = 0.05) among treatments at the same imbibition time. Error bars indicate ± SE of mean (*n* = 4). For additional explanations, please see **Figure 1**.

The transcription of NADPH oxidase gene (*NtRBOH*), involved in H_2_O_2_ production, was markedly upregulated by priming with GA (**Figure 11**), and was reversed by the addition of DPI in GA + DPI. Conversely, there was only weak enhancement or decrease with H_2_O_2_ or Uni compared with H_2_O, as well as H_2_O_2_ + Uni. *NtICL* was obviously upregulated by exogenous H_2_O_2_ or GA, especially H_2_O_2_ treatment, the *NtICL* expression was 10-fold of H_2_O at 0 h. Meanwhile, Uni kept the same level as H_2_O but DPI was significantly lower than H_2_O at the end of priming (**Figure 11D**, 0 h). The expression of *NtICL* in H_2_O_2_ + Uni was significantly higher than in H_2_O at 0 h and 24 h, and it was not found in GA + DPI.

## Discussion

Gibberellin acid was crucial for seed germination, and the seed germination ability was also related to the accumulation of H_2_O_2_ to a critical level (Weiss and Ori, 2007; Huang et al., 2017). In the present study, H_2_O_2_ and GA accelerated seeds to germinate 2 days in advance. While DPI and Uni, which are considered as generation inhibitors of H_2_O_2_ and GA, respectively, significantly delayed the germination by 2 and 1 days, respectively (**Figure 1** and **Supplementary Figure S2**). Meanwhile, the GP of DPI and Uni was obviously decreased, whereas the GP of DPI (43%) was slightly higher than that of Uni (35%). These results suggest that both GA and H_2_O_2_ are essential for seed germination and to guarantee germination speed and percentage. Similar results were found in flixweed (*Descurainia sophia* L.) and rice seeds; 10 μM uniconazole completely inhibited flixweed seed germination, and rice seed germination was inhibited by 10 μM DPI (Li et al., 2005; Ye and Zhang, 2012). Further evidence was presented in DPI + Uni treatment in which seeds barely germinated during the whole germination progress (**Supplementary Figure S1**). In addition, with H_2_O_2_ + Uni or GA + DPI treatment, seeds germinated 1 day in advance and the GP reached the same high level as with H_2_O_2_ + DPI or GA + Uni (**Supplementary Figure S1**). Furthermore, exogenous GA significantly increased the endogenous H_2_O_2_ content but Uni inhibited H_2_O_2_ synthesis (**Figure 2**). Likewise, exogenous H_2_O_2_ induced endogenous GA synthesis but DPI decreased the GA content (**Figures 4C,D**). These results all led to the preliminary conclusion that GA and H_2_O_2_ may mutually induce and have a synergistic effect on seed germination.

It is well known that ABA and GA are essential regulation factors in seed dormancy and germination (Toh et al., 2008; Stamm et al., 2012; Maarouf et al., 2015), and the antagonistic crosstalk between GA and ABA plays a pivotal role in the modulation of seed germination (Piskurewicz et al., 2008; Liu et al., 2016). Studies on numerous species have demonstrated that H_2_O_2_ promotes seed germination by triggering GA biosynthesis and ABA catabolism (Liu et al., 2010). DPI or Uni priming obviously increased the ABA content and the ABA/GA ratio (**Figures 4A,B** and **Supplementary Figure S3**), and decreased endogenous H_2_O_2_ content at the end of priming (**Figure 2**). These may be the reasons for intense reduction in the germination speed and the GP resulting from DPI or Uni. Arabidopsis seeds have evolved a mechanism to regulate the abundance of the antagonistically acting hormones ABA and GA before germination. GA increased synthesis arising from diminished ABA by downregulating *AtNCED9* and upregulating *AtCYP707A1* and *AtCYP707A2* (Topham et al., 2017). The mechanism also appeared in this study, and H_2_O_2_ and GA significantly reduced the ABA content at 24 h of imbibition and obviously increased the endogenous GA content at 0 and 24 h, and kept a lower level of the ABA/GA ratio during imbibition (**Supplementary Figure S3**). Exogenous GA reduced the expression of the ABA biosynthesis genes: *NtNCED1* from 0 to 48 h of imbibition, and *NtNCED3* (**Figure 5**) and *NtABI5* at 24 h (**Figure 7**). H_2_O_2_ mainly downregulated the transcription level of *NtNCED3* (**Figure 5**). Concomitantly, at 0 h of imbibition, ABA metabolic genes were highly upregulated by exogenous GA (12∼17-fold of H_2_O treatment), followed by H_2_O_2_ (9∼14-fold of H_2_O treatment) (**Figure 6**). However, DPI did not affect the expression of *NtCYP707A1* and *NtCYP707A2*. This result may hint that H_2_O_2_ affected *NtCPY707A* through GA rather than directly affecting them. Plenty of studies have shown that H_2_O_2_ affects GA content by upregulating synthetic gene and downregulating catabolism gene during seed germination (Liu et al., 2010; Gomes and Garcia, 2013). Further evidence has shown that H_2_O_2_ markedly upregulated *NtGA20_OX_2* (50-fold of H_2_O at 0 h) and *NtGA3_OX_2* (10-fold of H_2_O at 0 h) (**Figure 8**) from 0 to 48 h during imbibition, and downregulated *NtGA2_OX_1* (**Figure 9**). Notably, exogenous GA enhanced the production of endogenous GA by upregulating *NtGA20_OX_2* (**Figure 8**). This seems to be a self-induced feedback regulation. On the whole, these results suggest that there is a crosstalk between GA and H_2_O_2_ in mediating the homeostasis of ABA and GA.

On the other hand, the expression of GA signaling transduction genes *NtGID1* and *NtGID2* increased and the level of the seed germination inhibitor gene *NtRGL2* decreased after seed priming by GA or H_2_O_2_ (**Figure 10**), as different as possible from Uni or DPI. Cellular GA binds to the receptor GID, and the GID–GA complex interacts with DELLA to trigger the ubiquitination and destruction of RGL2, and induce seed germination (Ariizumi and Steber, 2007; Golldack et al., 2013). GA or H_2_O_2_ downregulated the expression of *NtABI3* and *NtABI5*, while Uni or DPI upregulated their expression. ABI3 and ABI5 were considered as the final downstream repressor of seed germination in the counterbalance of ABA and GA signals (Carles et al., 2002; Piskurewicz et al., 2008), and this was consistent with the RGL2-ABI5 model module that integrated the GA and ABA signaling pathways during seed germination (Liu et al., 2016). Another study has shown that H_2_O_2_ affects the ABA signal by inactivating ABI1 and ABI2 (Neill S. et al., 2002). These results suggest that there is a crosstalk between GA and H_2_O_2_ in mediating the signal transduction of ABA and GA.

Seed germination is a complex process controlled by many mechanisms, including the roles of GA and ABA (Toh et al., 2008), as well as radicle emergence (Guo et al., 2013), endosperm weakening (Zhang et al., 2014), and mobilization of storage reserves (Kelly et al., 2011; Han et al., 2013). During seed germination, lipolysis was followed by the gluconeogenesis pathway mainly conducting glucose production and sucrose used for seedling growth (Eckardt, 2005; Yan et al., 2014). For oilseeds, effective degradation of storage lipids is crucial for supporting respiratory substrates and energy generation, leading to successful seed germination and seedling establishment (Graham, 2008). Tobacco seeds contained higher lipid (30%) and lower sugar (10%) contents compared to the contents before priming (data are not shown), but the highest contents of soluble sugar in tobacco seeds was recorded at the end of priming (**Supplementary Figure S3**, 0 h). In addition, the change pattern of the ICL activity, which was involved in gluconeogenesis from lipid metabolism, was correlated with soluble sugar content during imbibition (**Figure 3**). Previous research conducted on oilseed species Arabidopsis (Meyer et al., 2012), sunflower (Raineri et al., 2016), and *Brassica rapa* (Gu et al., 2016) had also indicated that the levels of triacylglycerols, as components of lipids, decreased gradually during germination, suggesting that oilseed germination depends on oil breakdown and gluconeogenesis (Pritchard et al., 2002; Kelly et al., 2011).

Moreover, ABA and GAs have opposite roles in storage oil breakdown in the embryo (Penfield et al., 2006). Uni and DPI obviously increased the ABA content at 0 h of imbibition, and decreased the ICL activity at 0 and 24 h, significantly in DPI. ABA content increase accompanied with the reduction of ICL activity in Uni and DPI, this result was in agreement with the studies showed that exogenous ABA inhibited the activity of ICL in castor bean endosperm and *Brassica rapa* (Marriott and Northcote, 1977; Finkelstein and Lynch, 2000), and GA was able to reverse the inhibitory effect of ABA. However, it was also interesting to note that GA significantly decreased the endogenous ABA content at 24 and 48 h (**Figure 4**) but was ultimately ineffective in increasing the ICL activity (**Figure 3**); on the contrary, the ICL activity induced by H_2_O_2_ significantly increased at 0 and 24 h of imbibition without a decline in the ABA content (**Figure 3A**). Remarkably, exogenous GA obviously upregulated the expression of *NtRBOH*, encoded the NADPH oxidase biosynthesis genes and was involved in the generation of H_2_O_2_, triggering the accumulation of H_2_O_2_ content at 0 and 24 h of imbibition in tobacco seeds (**Figure 2**). It could be hypothesized that both exogenous GA and H_2_O_2_ increased the endogenous H_2_O_2_ content to enhance the expression of *NtICL* (**Figure 11**) and promote ICL activity (**Figure 3**); only in the case of exogenous H_2_O_2_ priming, the expression of *NtICL* was reflected in a significant promotion of ICL activity. The ABA/GA ratio of Uni and DPI was obviously high at 0 and 24 h (**Supplementary Figure S3**), but the ICL activity of Uni and DPI was low at 0 and 24 h (**Figure 3**); the ABA/GA ratio of GA and H_2_O_2_ kept low levels at 0 and 24 h but the ICL activity of GA and H_2_O_2_ kept high levels at 0 and 24 h. The present results support the conclusion that not the contents of ABA and GA, but the low ABA/GA ratio and the high ROS contents were both responsible for enhancing ICL activity. Previous studies by Yanik and Donaldson (2005) showed that a very high concentration of H_2_O_2_ inactivates ICL and degrades its product, glyoxylate, when CAT is inactive in castor beans. Similar results were also confirmed by Jeon et al. (2011) who showed that ICL activity was induced by a reasonable concentration H_2_O_2_ in *Bradyrhizobium japonicum*, as was evident at both the transcriptional and translational levels. In addition, earlier results indicated that the amounts and activities of ROS scavenging enzymes were strongly downregulated in barley treated with GA, thus increasing the H_2_O_2_ concentration. Nevertheless, ABA had the opposite effects (Fath et al., 2001). Curiously, H_2_O_2_ treatment had a weak effect on the *NtRBOH* expression levels (**Figure 11A**). In *N. benthamiana*, RBOH localized in the plasma membrane (PM) and the Golgi apparatus. PM-localized RBOH oxidase has been identified as a major source of ROS (Lherminier et al., 2009). ROS accumulation preceded RBOHD transcript- and protein-upregulation, indicating that ROS resulted from the activation of a PM-resident pool of enzymes, and that enzymes newly addressed to the PM were inactive (Noirot et al., 2014). Subcellular trafficking from Golgi to the PM was a potential determinant of RBOH activity, and this process regulation has mainly been addressed by considering the variation of gene expression or other signaling molecules, such as nitric oxide (NO) (Noirot et al., 2014). Both NO and ROS are known to react with each other and a balanced production between intracellular ROS and NO has been shown to be critical to the hypersensitive response (Delledonne et al., 2001). Yun et al. (2011) found that NO mediated S-nitrosylation of AtRBOHD governs a negative feedback loop limiting the production of ROS. We hypothesized that there is also feedback regulation between ROS and *NtRBOH*; H_2_O_2_ suppresses *NtRBOH* expression through other signaling when H_2_O_2_ is sufficient in the cell. Signaling activities of RBOH-derived ROS are probably also modulated by other ROS sources including xanthine oxidase, amine oxidase, and a cell wall peroxidase. It is possible that different stimuli activate specific H_2_O_2_-generating enzymes (Neill S.J. et al., 2002). Multiple source pathways of H_2_O_2_ may also be responsible for the inconsistency in H_2_O_2_ concentration and *NtRBOH* expression.

**FIGURE 12:**
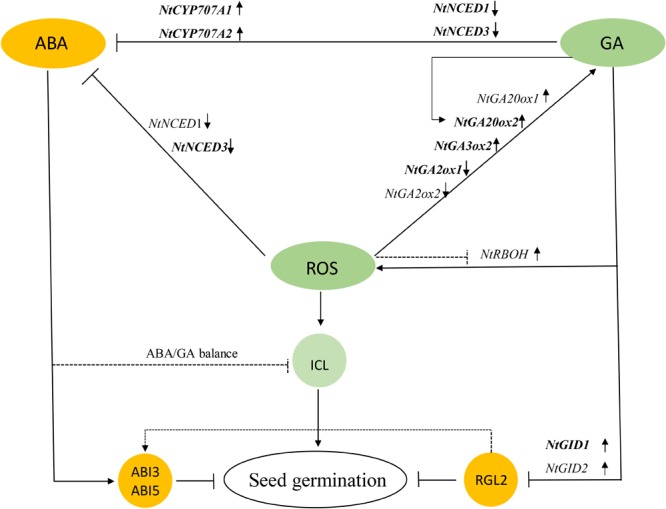
Model showing how GA, ROS, ABA, and ICL regulate seed germination. ROS led to increases in GA and decreases in ABA, where ABA inhibited seed germination and GA downregulated the DELLA Protein RGL2 suppressor to promote germination. ROS also enhanced ICL activity to directly mobilize storage reserves. GA feedback increased ROS, and on the other hand, GA inhibited the generation of ABA by downregulating ABA biosynthesis and upregulating ABA catabolism, indirectly stimulating seed germination; but a balance of these two hormones jointly controls the germination of tobacco seeds. GA, gibberellin acid; ROS, reactive oxygen species; ICL, isocitrate lyase.

The present results show that the application of DPI and Uni increased ABA content and decreased GA content, by regulating the corresponding gene expression and, eventually, delayed the germination time and reduced the GP. The experiment revealed that H_2_O_2_ and GA were essential for tobacco seed germination mainly because of downregulating the ABA/GA ratio and inducing reserve composition mobilization to mutually promote seed germination. In addition, the potential role of ROS in seed germination was proposed that it affected the homeostasis and the signal transduction of GA and ABA by activating or inactivating gene expressions, eventually decreasing the ABA/GA ratio. ROS is also attributed to enhanced ICL activity at a higher concentration (**Figure 12**). However, in this work, the interaction effects between ROS, GA, and ABA were primary determined, and the underlined mechanisms need further study.

## Author Contributions

ZL conceived and designed the experiments. ZL, YG, YZ, CL, and DG performed the experiments. ZL and YG analyzed the data. All authors contributed reagents, materials, and analysis tools. ZL, YG, and YJG wrote the paper.

## Conflict of Interest Statement

The authors declare that the research was conducted in the absence of any commercial or financial relationships that could be construed as a potential conflict of interest.
