# Supplementary material for: Reactive Oxygen Species and Gibberellin Acid Mutual Induction to Regulate Tobacco Seed Germination
Source: Front Plant Sci. 2018 Oct 2;9:1279. doi: 10.3389/fpls.2018.01279 (PMC6190896; doi:10.3389/fpls.2018.01279)
Supplement: Supplementary file 1 [file Presentation_1.pdf]

Supplementary data

Reactive oxygen species and gibberellin acid mutual induction to regulate tobacco seed germination

Zhan Li[1], Yue Gao[1**], Yuchan Zhang[1], Cheng Lin[1], Dongting Gong[1], Yajing Guan[1*], Jin Hu[1]**

[1] Seed Science Center, College of Agriculture and Biotechnology, Zhejiang University, Hangzhou 310058, China

Table S1. Real-time PCR primers used for genes expression analysis

Gene Name	Forward	Reverse
NtABI3	GAGTATCAGACCATGGAATCTGC	TTCCATCGCGGAGAATTG
NtABI5	CGCAAAAGGCGACTAACAA	ACACATCAAGGGCAACTCAA
NtGA20ox1	TTGCCACTTTCTGAAAAGCA	CTGGCATAACCACAATGCTC
NtGA20ox2	AATTCATATGGCCGGAAGG	GGAGGGACATGTAATGGAGGT
NtGA3ox2	TGGAAAAACTAGCCGGAAGA	GCCCATTTCATATCGTCCTTAC
NtGA2ox1	TCATTGTAAACTTCACCAAGCAA	AAATGGTCAATGCCAGGTTT
NtGA2ox2	TTGGAGGACCACCATTGAGT	CAAGCTGTCTTGATCCCCTTT
NtGID1	TTGCCTTCCTAGTTGCTGTG	CAGAACAGACACTTCTGAAATAGCA
NtGID2	ACCTCTGGCCACTCTCAAAG	CGGAAGTTGGAGTCGAAGAA
NtRGL2	GCAGTGACGCAGTCCAAA	TGTCAATTTATAACCATCACACGA
NtNCED1	TGGCATCTACAACTCCTGCTT	AGTCTCTTGAAGATGGCATTGAT
NtNCED3	TGGCATCTACAACTCCTGCTT	AGTCTCTTGAAGATGGCATTGAT
NtCYP707A1	GGAATTCAGTATGGGCCATTT	GGGATATAGCTTAATGGGCAGA
NtCYP707A2	TGTATGCCATATGCATCTAAGGTT	CTTGGAAACCAAGGCACAA
NtRBOH	CTGTTTTCTCCGAGGTTTGC	CAGCTCTGAGGAGTCCACTTTT
NtICL	AGCCCATGGAGTTGACACAT	TCTATCATAGTAATTAGCACCAGACC
Actin	CTATTCTCCGCTTTGGACTTGGCA	ACCTGCTGGAAGGTGCTGAGGGAA

**These authors contributed equally to this work.

*Corresponding author. Tel.: +86 57188982318; fax: +86 57188982318; E-mail address: vcguan@zju.edu.cn

Table S2. Seed vigor and viability of Yunyan 97 during seed germination

Treatments	GP			GI			MGT		
	2	5	7	2	5	7	2	5	7
H_2O	0.0±0.0* b	86.0±3.0 c	91.0±1.0 c	0.0±0.0 b	20.4±0.7 d	21.1±0.3 d	——	4.3±0.0 b	4.3±0.0 a
H_2O_2	5.0±1.0 a	92.0±2.0 a	95.0±1.0 a	2.5±0.5 a	24.3±0.9 b	24.8±0.8 b	2.0±0.0	3.9±0.0 d	3.9±0.1 c
Uni	0.0±0.0 b	0.0±0.0 d	31.7±2.1 e	0.0±0.0 b	0.0±0.0 e	5.3±0.4 f	——	—— —	0.4±0.2 g
H_2O_2+Uni	0.0±0.0 b	92.0±1.7 a	92.3±1.2 bc	0.0±0.0 b	22.3±0.4 c	22.4±0.3 c	——	4.2±0.0 c	4.2±0.0 ab
GA	5.7+1.2 a	91.0±1.0 ab	94.3±1.2 a	2.8±0.6 a	25.4±0.5 a	26.0±0.5 a	2.0±0.0	3.7±0.0 f	3.6±0.1 de
DPI	0.0±0.0 b	2.3±1.2 d	42.0±1.0 d	0.0±0.0 b	0.5±0.2 e	7.0±0.2 e	——	5.0±0.0 a	0.7±0.0 f
GA+DPI	0.0±0.0 b	91.7±1.5 a	94.7±1.5 a	0.0±0.0 b	22.0±0.4 c	22.5±0.4 c	——	4.2±0.0 bc	4.1±0.0 b
Uni+DPI	0.0±0.0 b	0.0±0.0 d	0.0±0.0 f	0.0±0.0 b	0.0±0.0 e	0.0±0.0 g	——	—— —	—— —
H_2O_2+DPI	0.0±0.0 b	89.0±1.0 b	94.0±1.0 ab	0.0±0.0 b	24.0±0.4 b	24.8±0.3 b	——	3.8±0.0 e	3.7±0.1 d
GA+Uni	5.7±1.2 a	90.7±1.2 ab	94.7±0.6 a	2.8±0.6 a	25.6±0.5 a	26.2±0.4 a	2.0±0.0	3.7±0.0 f	3.6±0.0 e

*Values were mean ± SE (n = 4). Different small letter (s) following the values indicated significant difference (LSD, α=0.05) during seed development. GP: germination percentage, GI: germination index, MGT: mean germination time (GP=100% × G_T/100, where G_T is the total germination seeds on the 2nd, 5th and 7th day, respectively; GI = ∑ (Gt/Tt), MGT =∑ (Gt×Tt) /∑Gt, where Gt is the number of new germinated seeds in time Tt, 2nd, 5th and 7th day.

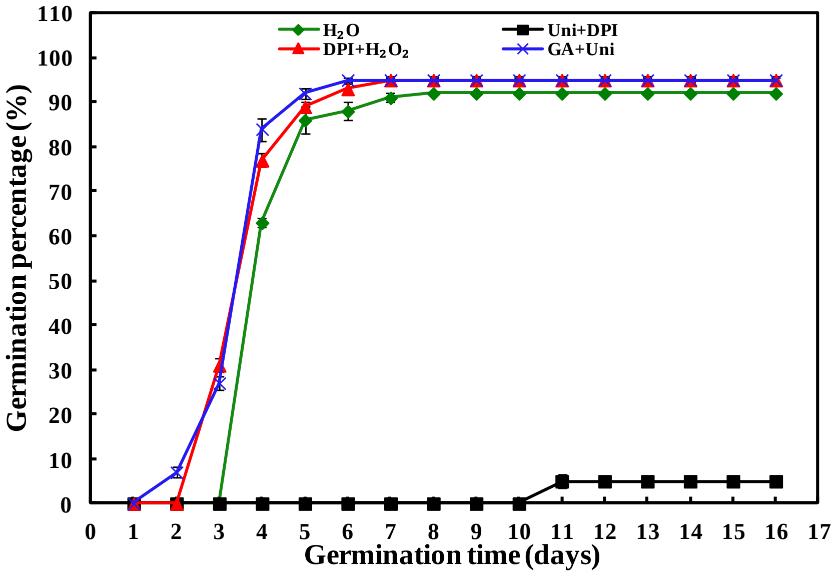

Fig. S1. GA or H_2O_2 was essential for seed germination. GA: gibberellin acid; H_2O_2: hydrogen peroxide ; Uni: uniconazole, GA synthesis inhibitor; DPI: diphenyliodonium, H_2O_2 scavenger. H_2O: hydropriming (water priming); Uni+ DPI: 30 µM Uni and 100 µM DPI combination priming. DPI+ H_2O_2: 50 mM H_2O_2 and 100µM DPI combination priming; GA+ Uni: 10 mM GA and 30 µM Uni combination priming. Vertical bars above mean indicated standard error of six replicates of 50 seeds each for each treatment. Values were mean ± SE (n = 6).

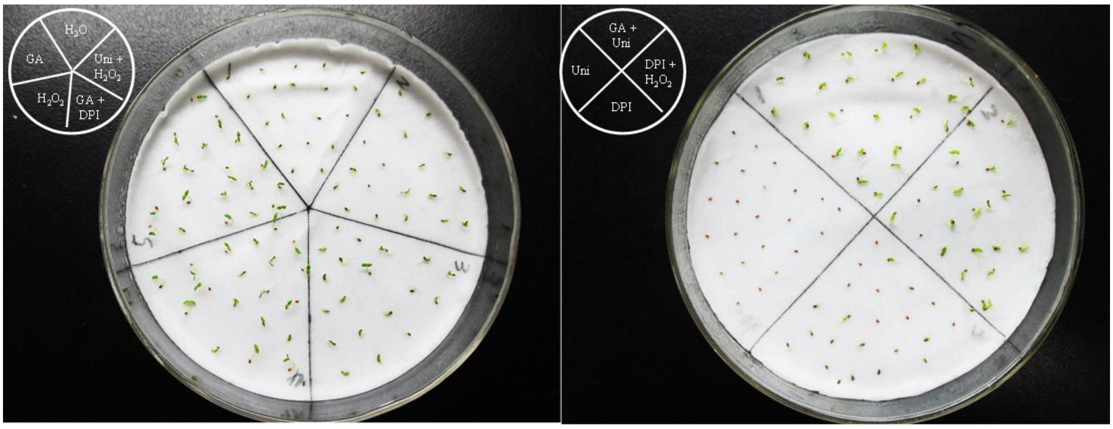

Fig. S2. Seed germination of different treatments on the fifth day after sowing. GA: gibberellin acid; H_2O_2: hydrogen peroxide ; Uni: uniconazole, GA synthesis inhibitor; DPI: diphenyliodonium, H_2O_2 scavenger. H_2O: hydropriming (water priming); Uni+ DPI: 30 µM Uni and 100 µM DPI combination priming. DPI+ H_2O_2: 50 mM H_2O_2 and 100µM DPI combination priming; GA+ Uni: 10 mM GA and 1 mM Uni combination priming.

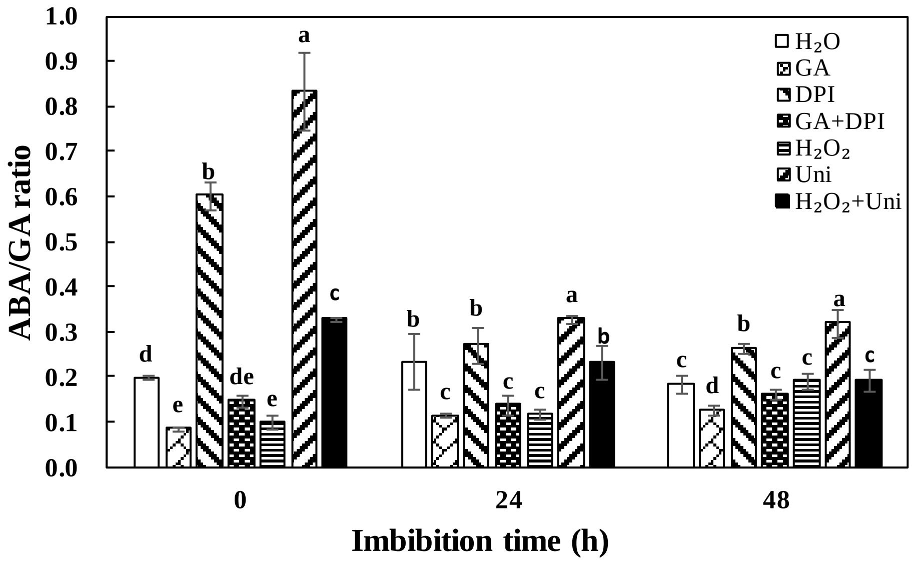

Fig. S3. Changes of ABA/GA_3 ratio during seed imbibition in response to different treatments. Seed were collected respectively at 0, 24 and 48 h during imbibition, and four replications for each treatment at each sampling time were used. Different small letter (s) on the top of the bars indicated significant differences (LSD, α=0.05) among treatments at same imbibition time. Error bars indicated ± SE of mean (n = 4). For additional explanations, please see Fig.1.

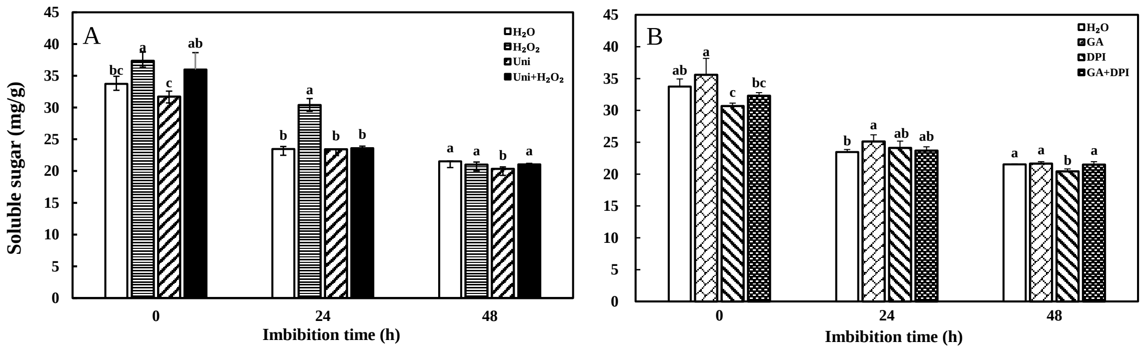

Fig. S4. Changes of soluble sugar during seed imbibition in response to different treatments. Seed were collected respectively at 0, 24 and 48 h during imbibition, and four replications for each treatment at each sampling time were used. Different small letter (s) on the top of the bars indicated significant differences (LSD, α=0.05) among treatments at same imbibition time. Error bars indicated ± SE of mean (n = 4). For additional explanations, please see Fig.1.
